# Assessment of joint line obliquity and its related frontal deformity using long-standing radiographs

**DOI:** 10.1016/j.jor.2023.04.014

**Published:** 2023-04-25

**Authors:** Tianshun Xie, Hugo C. van der Veen, Inge van den Akker-Scheek, Reinoud W. Brouwer

**Affiliations:** a Department of Orthopaedic Surgery, University of Groningen, University Medical Centre Groningen, P.O. Box 30.001, 9700 RB, Groningen, the Netherlands; b Department of Orthopaedic Surgery, Martini Hospital, P.O. Box 30.0331, 9700 RM, Groningen, the Netherlands

**Keywords:** Joint line obliquity, Frontal deformity, Long-standing radiograph, Bipedal distance, Osteoarthritis grade, High tibial osteotomy

## Abstract

**Purpose:**

To investigate how radiographic techniques and osteoarthritis grade influence measurements of knee joint line obliquity (KJLO) and KJLO-related frontal deformity, and to propose preferable KJLO measurement methods.

**Methods:**

Forty patients with symptomatic medial knee osteoarthritis indicated for high tibial osteotomy were assessed. Measurements were compared between single-leg and double-leg standing radiographs for KJLO measurement methods including joint line orientation angle by femoral condyles (JLOAF), joint line orientation angle by middle knee joint space (JLOAM), joint line orientation angle by tibial plateau (JLOAT), Mikulicz joint line angle (MJLA) and medial proximal tibial angle (MPTA), as well as KJLO-related frontal deformity parameters including joint line convergence angle (JLCA), knee ankle joint angle (KAJA) and hip-knee-ankle angle (HKA). Influences of bipedal distance in double-leg standing and osteoarthritis grade on the above measurements were analysed. Measurement reliability was evaluated by intraclass correlation coefficient.

**Results:**

From single-leg to double-leg standing radiographs MPTA and KAJA did not change significantly, whereas the other measurements showed significant changes: JLOAF, JLOAM and JLOAT decreased 0.88°, 1.24° and 1.77°, MJLA and JLCA decreased 0.63° and 0.85°, and HKA increased 1.11° (p < 0.05). Bipedal distance in double-leg standing radiographs moderately correlated with JLOAF, JLOAM and JLOAT (r_p_ = −0.555, −0.574 and −0.549). Osteoarthritis grade moderately correlated with JLCA in single-leg and double-leg standing radiographs (r_s_ = 0.518 and 0.471). All measurements had at least good reliability.

**Conclusion:**

In long-standing radiographs, measurements of JLOAF, JLOAM, JLOAT, MJLA, JLCA and HKA are all influenced by single-leg/double-leg standing; JLOAF, JLOAM and JLOAT are also affected by bipedal distance in double-leg standing; and JLCA is affected by osteoarthritis grade. Knee joint obliquity as assessed by MPTA measurement is independent of single-leg/double-leg standing, bipedal distance or osteoarthritis grade, and has excellent measurement reliability. We therefore propose MPTA as the preferable KJLO measurement method for clinical practice and future research.

**Level of evidence:**

III, cross-sectional study.

## 1 Introduction

High tibial osteotomy is an effective treatment option for symptomatic medial knee osteoarthritis with tibial varus deformity.^1^ However, a postoperative suspected excessive knee joint line obliquity (KJLO) can be introduced in the frontal plane after this surgical treatment, which seems to result in inferior clinical outcomes.^2^, ^3^, ^4^

Five KJLO measurement methods are described in literature, including joint line orientation angle by femoral condyles (JLOAF), joint line orientation angle by middle knee joint space (JLOAM), joint line orientation angle by tibial plateau (JLOAT), Mikulicz joint line angle (MJLA) and medial proximal tibial angle (MPTA), of which the JLOAT is the most frequently used.^3^, ^4^, ^5^, ^6^, ^7^, ^8^, ^9^ Also, three different frontal deformity parameters, including joint line convergence angle (JLCA), knee ankle joint angle (KAJA) and hip-knee-ankle angle (HKA), are related to a postoperative suspected excessive KJLO in high tibial osteotomy, and as such important measurement entities.^9^, ^10^, ^11^ Anteroposterior long radiographs with single-leg and double-leg standing are performed to assess both KJLO and the three KJLO-related frontal deformity parameters, with great variability in the bipedal distance used in the double-leg standing radiographs.^5^^,^^9^, ^10^, ^11^, ^12^, ^13^ The medial knee osteoarthritis severity grade differs in patients when assessing the KJLO and KJLO-related frontal deformity parameters.^3^^,^^14^^,^^15^

How radiographic techniques and osteoarthritis grade influence the measurements of KJLO and KJLO-related frontal deformity is not fully understood. To the best of our knowledge, there is no published consensus on which KJLO measurement method should be used. Preferable KJLO measurement methods need to be identified for clinical usage and research purposes.

The aim of the present study is to investigate the influences of long single-leg and double-leg standing radiographs, bipedal distance in double-leg standing, and osteoarthritis grade on the measurements of KJLO and KJLO-related frontal deformity, and to propose preferable KJLO measurement methods.

## 2 Methods

### 2.1 Study design

Patient database from a published study was reviewed.^16^ This database included 298 patients with symptomatic medial knee osteoarthritis and varus lower limb alignment, who were indicated for a high tibial osteotomy. From this database we included 130 patients who had both a preoperative anteroposterior long single-leg as well as a double-leg standing radiograph.

**Fig. 1:**
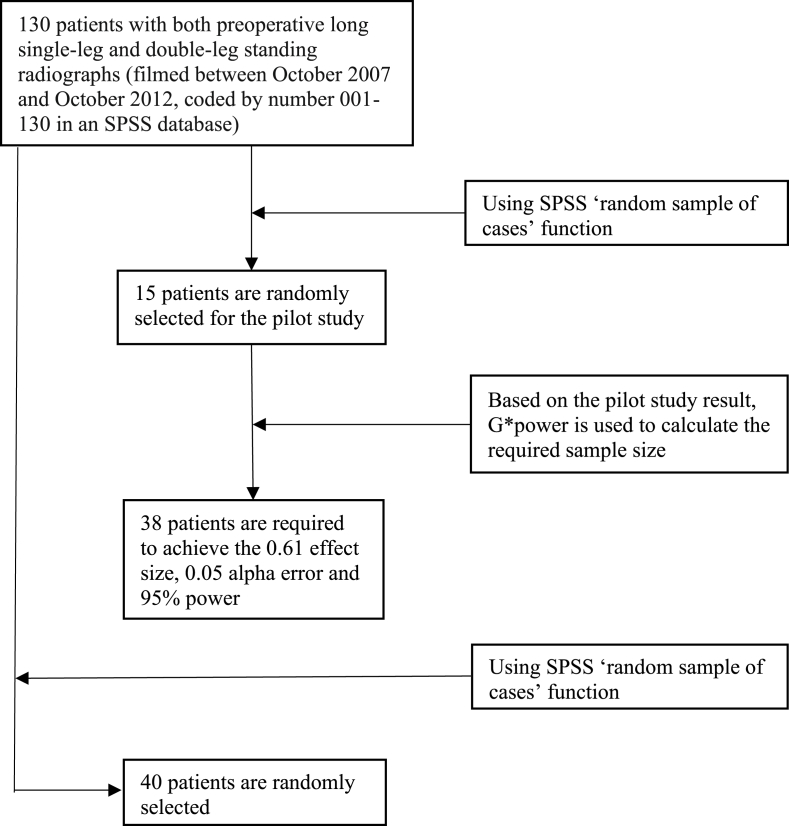
Flowchart of patient selection process.

The patient selection process is depicted in Fig. 1. Based on pilot study results, to detect a 1.66° mean measurement difference in JLOAT between the single-leg and the double-leg standing radiograph with a standard deviation of 2.73, a power of 95% and an alpha of 0.05, at least 38 patients were needed (G*power software, version 3.1.9.7). We randomly selected 40 patients (31 men and 9 women) with 80 anteroposterior long-standing radiographs.

The design and reporting of this study followed the STROBE (Strengthening the Reporting of Observational Studies in Epidemiology) checklist for cross-sectional studies.^17^ This study was approved by the ethics committee of our hospital (MEC no. 2022–005).

### 2.2 Long-standing radiographs

Anteroposterior long-standing radiographs were performed as follows: (1) single-leg standing: the patient stood barefoot on the affected leg, the affected knee in full extension and patella facing forward. The contralateral flexed knee was supported by a small box. The X-ray central beam targeted the affected knee centre and was perpendicular to the cassette at a distance of 1.5 m from the tube. (2) double-leg standing: the patient stood barefoot on double legs, both knees in full extension and patella facing forward. The X-ray central beam was targeted between the knees and was perpendicular to the cassette at a distance of 1.5 m from the tube.

### 2.3 Radiographic measurements

Picture Archiving and Communication System (PACS) software (Vue PACS, Philips, N·V.) was used for radiographic measurements. The minimum measurement differences that this software could determine were 0.01° for angle parameters and 0.01 cm for distance parameters.

Medial knee osteoarthritis grade was evaluated by the Kellgren-Lawrence classification.^18^ Two orthopaedic surgeons obtained the preoperative osteoarthritis grade in anteroposterior short-standing radiographs with the knee in full extension using paired-reading and sequence-known method.^16^

**Fig. 2:**
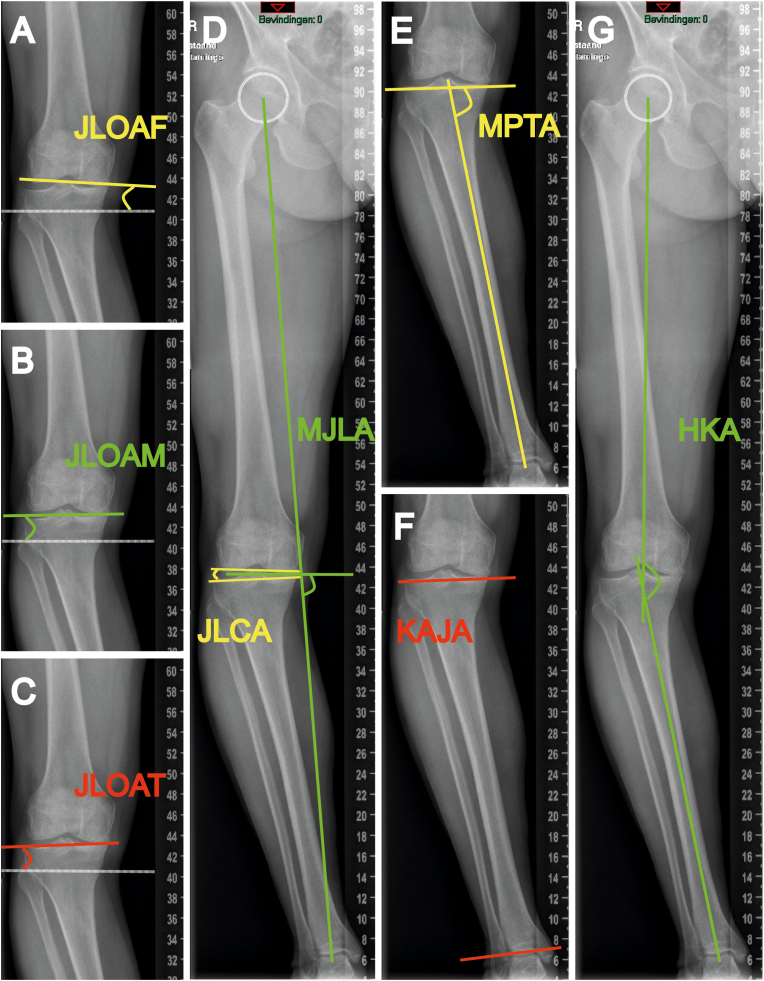
Measurements in anteroposterior long single-leg standing radiograph. Abbreviations: JLOAF, joint line orientation angle by femoral condyles; JLOAM, joint line orientation angle by middle knee joint space; JLOAT, joint line orientation angle by tibial plateau; JLCA, joint line convergence angle; MJLA, Mikulicz joint line angle; MPTA, medial proximal tibial angle; KAJA, knee ankle joint angle; HKA, hip-knee-ankle angle. Note: In this patient example, JLOAF, JLOAM, JLOAT, JLCA, MJLA, MPTA, KAJA and HKA are measured as −3.37°, 0.20°, 2.06°, 5.32°, 84.86°, 80.96°, −4.52° and 166.57°, respectively.

**Fig. 3:**
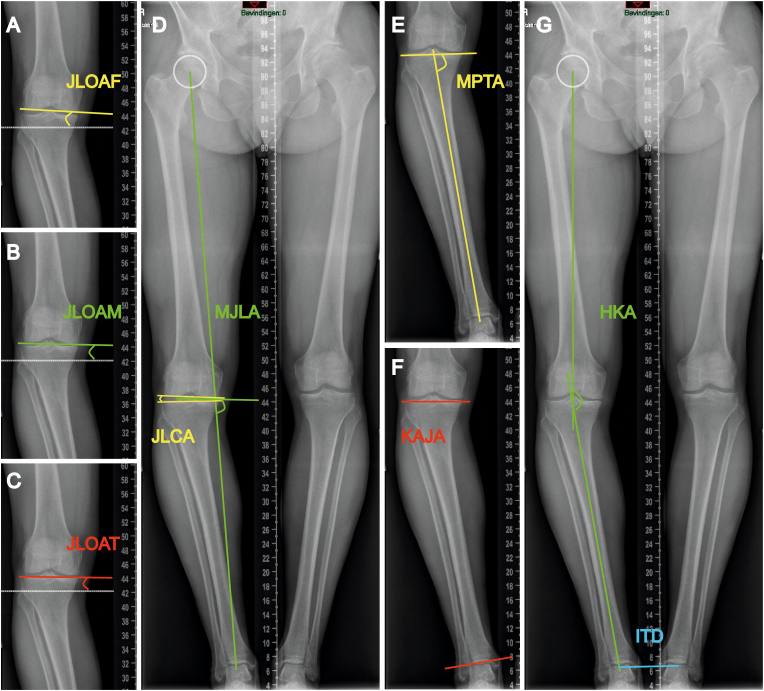
Measurements in anteroposterior long double-leg standing radiograph. Abbreviations: JLOAF, joint line orientation angle by femoral condyles; JLOAM, joint line orientation angle by middle knee joint space; JLOAT, joint line orientation angle by tibial plateau; JLCA, joint line convergence angle; MJLA, Mikulicz joint line angle; MPTA, medial proximal tibial angle; KAJA, knee ankle joint angle; HKA, hip-knee-ankle angle; ITD, intertalar distance. Note: This radiograph is from the same patient as in Fig. 2; JLOAF, JLOAM, JLOAT, JLCA, MJLA, MPTA, KAJA, HKA and ITD are measured as −3.81°, −1.48°, −0.58°, 2.39°, 83.66°, 80.69°, −4.73°, 169.03° and 8.42 cm, respectively.

Measurements were performed as illustrated in the anteroposterior long single-leg standing radiograph (Fig. 2) and double-leg standing radiograph (Fig. 3) from the same patient, following these procedures.

(1) JLOAF: The angle between the tangential line of the femoral condyles and the ground line.^5^ This angle represented the KJLO (Fig. 2, Fig. 3A).
(2) JLOAM: The angle between the line that connected the midpoints of the medial and lateral knee joint space and the ground line.^4^ This angle represented the KJLO (Fig. 2, Fig. 3B).
(3) JLOAT: The angle between the tangential line of the tibial plateau and the ground line.^6^ This angle represented the KJLO (Fig. 2, Fig. 3C).
(4) JLCA: The angle between the tangential line of the femoral condyles and the tangential line of the tibial plateau.^19^^,^^20^ This angle represented the knee intra-articular deformity (Fig. 2, Fig. 3D).
(5) MJLA: The medial angle between the bisector line of the JLCA and the lower limb weight-bearing line (Mikulicz line).^7^ This angle represented the KJLO (Fig. 2, Fig. 3D).
(6) MPTA: The medial angle between the tangential line of the tibial plateau and the tibial mechanical axis.^19^ This angle represented the KJLO (Fig. 2, Fig. 3E).
(7) KAJA: The angle between the tangential line of the tibial plateau and the tangential line of the distal tibial articular surface.^10^ This angle represented the deformity relation between the knee and ankle joints (Fig. 2, Fig. 3F).
(8) HKA: The medial angle between the femoral mechanical axis and the tibial mechanical axis.^21^ This angle represented the global deformity of the lower limb (Fig. 2, Fig. 3G).
(9) Intertalar distance (ITD): The distance between the centres of both talar domes, representing the bipedal distance.^22^ (Fig. 3G)

For the measurements of JLOAF, JLOAM, JLOAT and KAJA, a positive value (+) indicated a medial opening angle and a negative value (−) indicated a lateral opening angle.

The above measurements were performed independently by two observers (TX and RWB), each observer blinded to the other observer's measurements. All measurements were performed twice at a three-week interval. Intraobserver and interobserver reliability was assessed using the intraclass correlation coefficient (ICC).

### 2.4 Preferable KJLO measurement method

A preferable KJLO measurement method should have (1) adequate measurement stability: this measurement method was not influenced by the long single-leg or double-leg standing radiographs used, the bipedal distance used in the double-leg standing radiograph, or the knee osteoarthritis grade; and (2) adequate measurement reliability: this measurement method had at least good intraobserver and interobserver reliability (ICCs ≥0.75).

### 2.5 Statistical analysis

All statistical analyses were conducted using SPSS software (version 25, IBM Corporation, NY, USA). Descriptive statistics were used to present demographic data of patients, like gender and age. The distribution of continuous data was checked by Shapiro-Wilk test and Q-Q plots. Normally distributed data were described by mean ± standard deviation. Paired t-tests were used to compare the KJLO and KJLO-related measurement data between the single-leg and double-leg standing radiographs. Pearson correlation coefficients were calculated to determine the correlations between the bipedal distance in the double-leg standing radiographs and the KJLO and KJLO-related measurement data. Spearman correlation coefficients were calculated to determine the correlations between the osteoarthritis grade and the KJLO and KJLO-related measurement data. ICCs (two-way mixed, absolute agreement) were calculated to determine intraobserver and interobserver measurement reliability.^23^ A p-value <0.05 was considered statistically significant.

### 2.6 Measurement reliability and correlation magnitude

Measurement reliability was graded in accordance with Koo's guideline.^23^ The ICCs <0.50, 0.50–0.75, 0.75–0.90, and >0.90 indicated poor, moderate, good, and excellent reliability, respectively. The interpretation of a correlation magnitude was in accordance with Schober's tutorial.^24^ Correlation coefficient values of 0.00–0.10, 0.10–0.39, 0.40–0.69, 0.70–0.89, and 0.90–1.00 indicated negligible, weak, moderate, strong, and very strong magnitude, respectively.

## 3 Results

### 3.1 Patient characteristics

Patients’ age at filming was 49.1 ± 8.3 years (range 24–65). The osteoarthritis grades of the medial knee compartment were Kellgren-Lawrence grade I in 13 knees, grade II in 18 knees, and grade III in 9 knees. Bipedal distance in the long double-leg standing radiographs was 13.89 ± 4.07 cm.

### 3.2 Single-leg versus double-leg standing

The KJLO measurements and KJLO-related frontal deformity parameters performed on the single-leg and double-leg standing radiographs are described in Table 1. Mean JLOAF differed by 0.88° on long single-leg compared to double-leg standing radiographs, mean JLOAM differed by 1.24°, mean JLOAT by 1.77°, mean MJLA by 0.63°, mean JLCA by 0.85°, and mean HKA differed by 1.11°.

**Table 1:** Single-leg versus double-leg standing radiograph.

	Single-leg standing radiograph		Double-leg standing radiograph		Measurement difference		P-value
Radiological parameters	mean	standard deviation	mean	standard deviation	mean	95% confidence interval	
JLOAF	−1.11°	2.41	−2.00°	2.15	0.88°	0.17°–1.60°	0.016*
JLOAM	0.76°	2.28	−0.48°	2.00	1.24°	0.52°–1.95°	0.001*
JLOAT	2.61°	2.60	0.85°	2.20	1.77°	1.12°–2.41°	<0.001*
MJLA	88.20°	1.75	87.57°	1.80	0.63°	0.37°–0.88°	<0.001*
MPTA	86.42°	2.49	86.13°	2.55	0.29°	−0.06°–0.64°	0.1
JLCA	3.49°	1.50	2.64°	1.27	0.85°	0.57°–1.13°	<0.001*
KAJA	−1.15°	3.59	−1.06°	3.73	−0.09°	−0.63°–0.46°	0.752
HKA	173.03°	3.07	174.14°	2.94	−1.11°	−1.38° to −0.84°	<0.001*

Statistical significance*.

Abbreviations: JLOAF, joint line orientation angle by femoral condyles; JLOAM, joint line orientation angle by middle knee joint space; JLOAT, joint line orientation angle by tibial plateau; MJLA, Mikulicz joint line angle; MPTA, medial proximal tibial angle; JLCA, joint line convergence angle; KAJA, knee ankle joint angle; HKA, hip-knee-ankle angle.

### 3.3 Bipedal distance

The bipedal distance (measured as ITD) in the double-leg standing radiographs and the correlations with the KJLO measurements and KJLO-related frontal deformity parameters are presented in Table 2. There were moderate negative correlations between ITD and JLOAF, JLOAM and JLOAT.

**Table 2:** Bipedal distance and osteoarthritis grade.

	Bipedal distance		Osteoarthritis grade			
	Double-leg standing radiograph		Single-leg standing radiograph		Double-leg standing radiograph	
**Radiological parameters**	Coefficient (r_p_)	p-value	Coefficient (r_s_)	p-value	Coefficient (r_s_)	p-value
JLOAF	−0.555	<0.001*	−0.012	0.942	−0.146	0.368
JLOAM	−0.574	<0.001*	0.091	0.575	0.034	0.835
JLOAT	−0.549	<0.001*	0.181	0.264	0.122	0.454
MJLA	−0.002	0.992	0.060	0.714	0.105	0.519
MPTA	0.019	0.908	−0.077	0.637	−0.004	0.980
JLCA	0.062	0.702	0.518	<0.001*	0.471	0.002*
KAJA	0.036	0.826	−0.001	0.994	−0.013	0.937
HKA	0.017	0.915	−0.326	0.040*	−0.316	0.047*

Statistical significance*.

Abbreviations: r_p,_ Pearson correlation coefficient; r_s,_ Spearman correlation coefficient; JLOAF, joint line orientation angle by femoral condyles; JLOAM, joint line orientation angle by middle knee joint space; JLOAT, joint line orientation angle by tibial plateau; MJLA, Mikulicz joint line angle; MPTA, medial proximal tibial angle; JLCA, joint line convergence angle; KAJA, knee ankle joint angle; HKA, hip-knee-ankle angle.

Note: Bipedal distance is evaluated by intertalar distance, osteoarthritis grade (I, II, III) is evaluated by Kellgren-Lawrence classification.

### 3.4 Osteoarthritis grade

Osteoarthritis grade (Kellgren-Lawrence I, II, III) and the correlations with the KJLO measurements and KJLO-related frontal deformity parameters are presented in Table 2. In single-leg standing radiographs, osteoarthritis grade had weak positive correlation with JLOAT and weak negative correlation with HKA. In double-leg standing radiographs, osteoarthritis grade had weak positive correlations with JLOAT and MJLA and weak negative correlations with JLOAF and HKA. Osteoarthritis grade correlated moderately positively with JLCA in single-leg standing radiographs and in double-leg standing radiographs.

### 3.5 Measurement reliability

Intraobserver and interobserver reliability is described in Table 3. All measurements had at least good measurement reliability, with measurements JLOAF, JLOAT, MPTA, HKA and ITD having excellent intraobserver and interobserver reliability.

**Table 3:** Measurement reliability.

	Intraobserver ICCs	Interobserver ICCs
Single-leg standing radiograph		
JLOAF	0.96–0.99 (excellent)	0.93–0.98 (excellent)
JLOAM	0.96–0.99 (excellent)	0.93–0.99 (excellent)
JLOAT	0.97–0.99 (excellent)	0.91–0.98 (excellent)
MJLA	0.94–0.98 (excellent)	0.92–0.98 (excellent)
MPTA	0.96–0.99 (excellent)	0.95–0.99 (excellent)
JLCA	0.92–0.98 (excellent)	0.85–0.96 (good-to-excellent)
KAJA	0.85–0.96 (good to excellent)	0.84–0.95 (good-to-excellent)
HKA	0.99–1 (excellent)	0.95–0.99 (excellent)
**Double-leg standing radiograph**		
JLOAF	0.95–0.99 (excellent)	0.91–0.97 (excellent)
JLOAM	0.94–0.98 (excellent)	0.89–0.97 (good-to-excellent)
JLOAT	0.95–0.99 (excellent)	0.93–0.98 (excellent)
MJLA	0.93–0.98 (excellent)	0.88–0.97 (good-to-excellent)
MPTA	0.96–0.99 (excellent)	0.95–0.99 (excellent)
JLCA	0.92–0.98 (excellent)	0.85–0.95 (good-to-excellent)
KAJA	0.90–0.98 (good to excellent)	0.85–0.95 (good-to-excellent)
HKA	0.94–0.98 (excellent)	0.96–0.99 (excellent)
ITD	1 (excellent)	0.99–1 (excellent)

Abbreviations: ICCs, intraclass correlation coefficients; JLOAF, joint line orientation angle by femoral condyles; JLOAM, joint line orientation angle by middle knee joint space; JLOAT, joint line orientation angle by tibial plateau; MJLA, Mikulicz joint line angle; MPTA, medial proximal tibial angle; JLCA, joint line convergence angle; KAJA, knee ankle joint angle; HKA, hip-knee-ankle angle; ITD, intertalar distance.

Note: The ICCs are graded on 95% confidence interval. ICCs <0.50, 0.50–0.75, 0.75–0.90, and >0.90 indicated poor, moderate, good, and excellent reliability, respectively.^23^.

## 4 Discussion

The main finding of this study is that there is a significant difference in determining KJLO using JLOAF, JLOAM, JLOAT and MJLA between single-leg and double-leg standing radiographs, which is influenced by degree of loading and decreases in the double-leg standing radiograph. An increase in bipedal distance in double-leg standing radiographs results in lower KJLO using JLOAF, JLOAM and JLOAT, and a higher medial knee osteoarthritis grade correlates moderately with a more varus-aligned JLCA.

Among the five KJLO measurement methods and the three KJLO-related frontal deformity parameters, MPTA and KAJA were not influenced by the long single-leg or double-leg standing radiographs used. This is because the measurements of MPTA and KAJA depend on the tibial geometry, which should remain unchanged with the degree of weight-loading adjustment. Our finding on the influences of single-leg and double-leg standing on JLCA and HKA is consistent with previous research, even though there are differences: the present study finds a difference in JLCA of 0.85° and a difference in HKA of 1.11° when determined on single-leg and double-leg standing radiographs in patients with medial knee osteoarthritis (Kellgren-Lawrence I, II, III) and varus alignment, whereas Yazdanpanah et al. report a difference in JLCA of 0.42° JLCA and in HKA of 0.76° in patients with knee osteoarthritis and varus/valgus alignment,^25^ and Bardot et al. report a difference in JLCA of 0.8° and in HKA of 1.92° in patients with medial knee osteoarthritis (Ahlbäck grades I, II) and tibial-originating varus deformity.^26^

An increase in bipedal distance results in lower JLOAF, JLOAM and JLOAT in long double-leg standing radiographs. Previous research assessed the JLOAT measurement in long double-leg standing radiographs of patients who underwent total knee replacement, and a change of 3.7° JLOAT per 10-cm bipedal distance was reported.^22^ Referencing the ground line during the measurement procedure may be the reason why JLOAF, JLOAM and JLOAT are all affected by bipedal distance in double-leg standing radiographs. Hence for studies that measure JLOAF, JLOAM and JLOAT in double-leg standing radiographs, a feet-together position or a footplate should be used to fix the bipedal distance.^19^ The bipedal distance in the double-leg standing radiographs should at least be reported: JLOAM and JLOAT have been used to determine the acceptable KJLO upper limits in other studies,^2^^,^^8^^,^^27^ but the determined upper limit values may not be accurate as the bipedal distance used at filming was not described in these studies.

Medial knee osteoarthritis grade does not affect KJLO measurements but does influence the KJLO-related frontal deformity parameter of JLCA. Our finding indicates that a higher medial knee osteoarthritis grade (Kellgren-Lawrence I, II, III) moderately relates to a higher magnitude of knee intra-articular varus deformity illustrated by a higher JLCA degree. Also, the present study finds a weak correlation between medial knee osteoarthritis grade and the global deformity parameter of HKA, in contrast to a study on the correlation magnitude: Brouwer et al. assessed the HKA measurement in long double-leg standing radiographs of patients with medial knee osteoarthritis (Ahlbäck grades I, II, III), and reported a strong correlation between osteoarthritis grade and HKA (r = 0.75).^28^ There are differences between the present and previous studies, including patient numbers, osteoarthritis grade classification system used, and whether or not lateral fluoroscopy is used to ensure a 100% anteroposterior projection, which may affect the correlation magnitude of osteoarthritis grade and HKA.

Although all measurements have shown at least good reliability, the reliability of KAJA appears inferior to those radiographic parameters with both excellent intraobserver and interobserver reliability. As a novel radiographic parameter, KAJA is used much less frequently than the other parameters by our observers in daily clinical practice. A reasonable speculation is that a lack of observers’ past measurement experience may negatively influence the determined measurement reliability of this novel radiographic parameter.

According to the predefined criteria, MPTA should be the preferable KJLO measurement method, as it has both adequate measurement stability and reliability. JLOAF, JLOAM, JLOAT and MJLA lack measurement stability, which restricts comparison of KJLO measurement results between studies using long single-leg and double-leg standing radiographs. The lack of measurement stability in JLOAF, JLOAM and JLOAT also hampers the acceptable KJLO upper-limit determination in studies using nonstandardised bipedal distance in double-leg standing radiographs. In addition to the predefined criteria, based on our current measurement experience we find that the measurement procedure of MJLA is more complicated and time-consuming than the other four KJLO measurement methods, which also limits the usage of MJLA.

To predict a postoperative suspected excessive KJLO, using KAJA could have more advantages than JLCA and HKA. This is because KAJA can be performed regardless of the long single-leg or double-leg standing radiographs used. Also, KAJA is not affected by osteoarthritis grade. When measuring JLCA and HKA, the long-standing radiograph used should be well-described.

The strength of this study is that the outcome helps fill the knowledge gap on how to assess KJLO and its related frontal deformity using long-standing radiographs. Choosing a measurement method without adequate stability may explain the conflicting evidence on the relation between KJLO and clinical outcomes in literature.^2^^,^^9^ We therefore propose a preferable KJLO measurement method that can be used to determine the actual relation between KJLO and clinical outcomes.

As a limitation, although all anteroposterior long-standing radiographs were made with knee in full extension and patella in forward position, the lateral fluoroscopic control that secures a 100% anteroposterior image without rotation was not applied. As a consequence, some rotation variations could be present at filming, which may affect the radiographic measurements in this study.

## 5 Conclusion

In long-standing radiographs, measurements of JLOAF, JLOAM, JLOAT, MJLA, JLCA and HKA are all influenced by single-leg/double-leg standing; JLOAF, JLOAM and JLOAT are also affected by bipedal distance in double-leg standing; and JLCA is affected by osteoarthritis grade. Knee joint obliquity as assessed by MPTA measurement is independent of single-leg/double-leg standing, bipedal distance or osteoarthritis grade, and has excellent measurement reliability. We therefore propose MPTA as the preferable KJLO measurement method for clinical practice and future research.

## Funding

This research did not receive any specific grant from funding agencies in the public, commercial or non-for-profit sectors.

## Informed consent

Not applicable.

## Institutional ethical committee approval

This study was approved by the Ethics Committee of Martini Hospital Groningen, The Netherlands (MEC no. 2022–005).

## Authors’ contribution

**TX:** Conceptualization, Data curation, Formal analysis, Writing-Original Draft; **HV:** Conceptualization, Writing-Review and Editing; **IA:** Conceptualization, Writing-Review and Editing; **RB:** Conceptualization, Data curation, Supervision, Writing-Review and Editing. All authors approved the final manuscript.

## Declaration of competing interest

None.
